# From Nature to Remediation: Biomaterials for Malachite Green Retention and Degradation

**DOI:** 10.3390/ma18184374

**Published:** 2025-09-19

**Authors:** Raluca Florenta Doroftei, Mihaela Silion, Daniela Ioniță, Andrei Dascalu, Florin Nedeff, Ana-Maria Georgescu, Ana-Maria Rosu, Diana Mirila, Ileana-Denisa Nistor

**Affiliations:** 1 School of Doctoral Studies, “Vasile Alecsandri” University of Bacau, 157 Calea Marasesti Street, 600115 Bacau, Romania; ralucadoroftei@yahoo.com; 2 Physics of Polymers and Polymeric Materials, “Petru Poni” Institute of Macromolecular Chemistry, 41A Grigore Ghica Voda Alley, 700487 Iasi, Romania; silion.mihaela@icmpp.ro (M.S.); ionita.daniela@icmpp.ro (D.I.); idascalu@icmpp.ro (A.D.); 3 Department of Environmental Engineering, Mechanical Engineering and Agritourism, Faculty of Engineering, “Vasile Alecsandri” University of Bacau, 157 Calea Marasesti Street, 600115 Bacau, Romania; florin_nedeff@ub.ro; 4 Department of Chemical and Food Engineering, Faculty of Engineering, “Vasile Alecsandri” University of Bacau, 157 Calea Marasesti Street, 600115 Bacau, Romania; ana.rosu@ub.ro

**Keywords:** biomaterials, fish, adsorption, catalytic ozonation, malachite green

## Abstract

The increasing presence of synthetic dyes in aquatic environments presents a serious threat to ecosystems and human health. This study investigates the potential of natural biomaterials, specifically fish-derived components extracted from *Cyprinus carpio* (fish bladder and fish scales), for the simultaneous retention and degradation of a potentially toxic dye: Malachite Green (MG). The biomaterials were characterized using X-ray diffraction (XRD), Fourier-transform infrared (FTIR) spectroscopy, scanning electron microscopy (SEM), energy-dispersive X-ray (EDX) spectroscopy, thermogravimetric analysis (TGA), and high-performance liquid chromatography with mass spectrometry detection (HPLC-MS) for degradation monitoring. Batch adsorption experiments were conducted under varying biomaterial dosage, contact time and pH. Results demonstrated that all tested biomaterials exhibited significant adsorption capacities, with fish scales (FS) achieving a maximum removal efficiency of 91.2%, and fish bladder (FB) reaching 82% under optimal conditions. In catalytic ozonation tests, the fish scales impregnated with vanadium (FS-V) catalyst demonstrated significantly higher degradation efficiency, reaching 63.84% at an ozone flow rate of 0.5 g O_3_·h^−1^. The comparative analysis highlights the multifunctionality of these eco-friendly biomaterials, offering both pollutant capture and partial degradation. These findings suggest that low-cost, naturally derived biomaterials can serve as effective alternatives to synthetic adsorbents in water treatment applications, contributing to sustainable environmental remediation strategies.

## 1. Introduction

**Figure 1:**
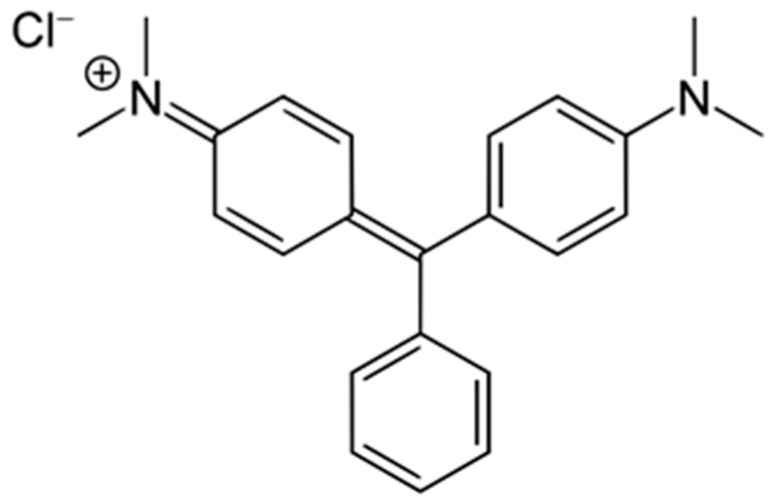
Chemical structure of MG.

The contamination of aquatic environments with synthetic dyes has become a critical environmental issue, driven by industrial discharge, agricultural runoff, and aquaculture practices. Among these pollutants, MG, presented in Figure 1, is particularly concerned due to its persistence, toxicity, and resistance to biodegradation [1,2].

MG, a cationic triphenylmethane dye, strongly binds to negatively charged surfaces, and requires oxidative or hydrolytic degradation. The complete removal and degradation of MG demands multifunctional materials capable of both adsorption and catalytic transformation.

MG is commonly used in agriculture and aquaculture due to its strong antibacterial, antifungal, antiparasitic, and coloring properties. It is also applied in the paper, leather, and silk industries, as well as in histological staining, particularly as a blue-green counterstain for safranin in the *Gimenez* technique [3].

MG is environmentally hazardous, accumulating in aquatic sediments and potentially leading to the decline or extinction of fish species due to its toxicity. It is cytotoxic to mammalian cells and acts as a tumor-promoting agent [4]. In humans, it may exert carcinogenic, mutagenic, and teratogenic effects if it enters the food chain [5]. Although banned in several countries and not approved by the US Food and Drug Administration, MG remains widely used due to its low cost, availability, and high efficacy [6,7].

According to recent studies, MG concentrations in environmental samples typically range from 0.1 to 10 mg·L^−1^, as determined by LC-MS/MS methods. Despite its toxicity, MG is still detected in industrial effluents due to its widespread use and environmental persistence. Regulatory frameworks vary, but MG is not approved by the US FDA and is classified as a Class II health hazard, raising concerns about its presence [8].

Adsorption remains one of the most effective and economical methods for pollutant removal due to its simplicity, efficiency, and adaptability. Materials such as clays, activated carbons, and bioadsorbents have demonstrated high adsorption capacities for dyes. The adsorption process is influenced by surface area, pore structure, and the presence of functional groups that interact with pollutants via electrostatic attraction, hydrogen bonding, or π–π interactions [9,10,11]. Biomaterials derived from fish waste, such as scales, bones, and swim bladders, are gaining attention as sustainable adsorbents. Rich in hydroxyapatite and collagen, these materials offer functional groups (-COOH, -NH_2_) that facilitate pollutant binding [12,13]. Their valorization supports circular economic principles and reduces organic waste.

Catalytic ozonation is an advanced oxidation process (AOP) that enhances the degradation of refractory organic pollutants by generating reactive oxygen species (ROS) such as hydroxyl radicals. Unlike conventional ozonation, catalytic ozonation uses solid catalysts, often metal oxides or carbon-based materials, to accelerate ozone decomposition and improve mineralization efficiency [14,15,16,17]. Recent studies have demonstrated the effectiveness of metal oxide thin-film catalysts (e.g., Fe_2_O_3_, Co_3_O_4_) in structured catalytic ozonation reactors for dye detoxification, achieving superior kinetics compared to classical ozonation [18]. Moreover, carbon-based catalysts have shown promise in carbocatalytic ozonation, leveraging the synergy between ozone and surface-bound ROS [19].

Hybrid systems combining adsorption and ozonation have emerged as powerful tools for pollutant removal. These systems exploit the rapid adsorption of pollutants onto materials followed by catalytic ozonation for complete degradation. Such integrated approaches enhance efficiency, reduce ozone dosage, and minimize toxic byproducts [9,20,21].

Recent advances in photocatalysis have shown that modifying the surface chemistry and constructing heterostructures can significantly enhance the degradation efficiency of organic pollutants. Zhang et al. developed a TiO_2_-based photocatalyst derived from NH_2_-MIL-125(Ti), doped with palladium via a quenching process, which achieved 97% tetracycline degradation and 95% mineralization, due to improved charge separation and surface reactivity [22]. Similarly, MIL-101(Fe)/Bi_2_MoO_6_ heterostructures synthesized via solvothermal methods exhibited enhanced photocatalytic activity, removing 96.2% of tetracycline hydrochloride and enabling efficient nitrogen photofixation without sacrificial agents [23]. Furthermore, MIL-101(Fe)/WO_3_ hybrid photocatalysts designed through in situ immobilization demonstrated 93.8% degradation efficiency, driven primarily by photogenerated holes, and confirmed the effectiveness of S-scheme mechanisms in promoting charge separation and photostability [24].

Inspired by these strategies, our study investigates the functionalization of fish-derived biomaterials, such as swim bladders and scales, with vanadium, aiming to replicate similar catalytic enhancements. Vanadium, known for its redox versatility and ability to promote electron transfer, is introduced to create active sites that support both adsorption and photocatalytic degradation of organic contaminants. This dual-functionality approach combines the natural adsorption capacity of biosorbents with the catalytic benefits of transition metal doping, offering a sustainable and efficient solution for wastewater treatment.

Catalytic ozonation has emerged as an effective advanced oxidation process (AOP) for the degradation of synthetic dyes in wastewater, offering enhanced mineralization and color removal compared to ozonation alone. Hu et al. demonstrated that manganese ferrite (MnFe_2_O_4_) supported on carbon aerogel significantly improved the degradation of reactive dyes in textile effluents, achieving a 25% increase in COD removal and enabling effluent reuse without compromising fabric quality [25]. Similarly, Bilińska et al. reviewed homogeneous and heterogeneous catalytic ozonation mechanisms, emphasizing the role of hydroxyl radicals and novel nanostructured catalysts in decomposing persistent dye molecules [26]. In another study, Ali et al. used iron and manganese-loaded zeolites to remove Disperse Yellow-42 dye, achieving up to 73% dye removal and 79% COD reduction in real textile wastewater [27].

In addition to inorganic catalysts, biocatalysts, such as enzyme-functionalized supports or biochar derived from agricultural and marine waste, have shown promise in catalytic ozonation systems. These materials can enhance ozone decomposition and promote the generation of reactive oxygen species under mild conditions. For instance, laccase-immobilized biochar and peroxidase-based systems have been used to degrade dyes like methylene blue and reactive black 5, offering eco-friendly and selective catalytic pathways. Such biocatalytic approaches align well with the concept of using fish-derived biosorbents functionalized with vanadium, combining natural adsorption capacity with catalytic activity to improve the degradation of organic dyes in wastewater.

In line with the growing scientific interest in bio-adsorbents for the removal of synthetic dyes such as MG, Table 1 summarizes a selection of natural materials previously investigated for this purpose.

**Table 1:** Reported bio-adsorbents used for MG dye removal.

Material	Type	Reference
*Cordia africana* leaf	Bio-adsorbent	[28]
White clover	Bio-adsorbent	[29]
Raw tea waste (RTW) and iron coated tea waste (FeTW)	Bio-adsorbent	[30]
Java Plum Leaves Powder (JPLP)	Bio-adsorbent	[31]
*Russula delica* mushroom/bentonite clay (RDBNC)	Bio-adsorbent	[32]
Lignocellulosic rice straw fibers	Bio-adsorbent	[33]
*Themeda arundinacea*	Bio-adsorbent	[34]
Marine alga *S. wightii*	Bio-adsorbent	[35]
Activated Clove Leaf (CL-KOH)	Bio-adsorbent	[36]

Although significant progress has been made in the development of bio-derived adsorbents, comparative investigations focusing exclusively on fish-based biomaterials remain underexplored. This study contributes to filling this gap by assessing the performance of two vanadium-impregnated biosorbents synthesized from *Cyprinus carpio*, fish bladder (FB-V) and FS-V, in the retention/degradation of MG under various environmental conditions. Vanadium, particularly in the form of vanadium pentoxide (V_2_O_5_), is a well-known transition metal oxide with strong photocatalytic properties. Its variable oxidation states (V^5+^/V^4+^) enable redox cycling, which facilitates the generation of ROS such as hydroxyl radicals (•OH) and superoxide anions (•O_2_^−^). These ROS are responsible for breaking down dye molecules into smaller, less toxic compounds or mineralizing them into CO_2_ and H_2_O [37,38]. Vanadium-based catalysts also exhibit visible light activity due to their suitable band gap (~2.3 eV), making them effective under ambient conditions. In this study, in the case of FS-V, vanadium likely enhances the oxidative degradation of MG by promoting electron transfer and ROS generation in synergy with ozone.

Furthermore, previous research has demonstrated the suitability of fish swim bladder as a precursor for porous carbon used in water defluoridation, reinforcing the environmental relevance and versatility of such biomaterials [39]. The incorporation of vanadium is investigated as a strategy to enhance catalytic ozonation, leveraging its redox flexibility and proven ability to activate ozone [40]. To date, most studies have concentrated on plant- or fungus-derived bioadsorbent, while applications involving fish-based materials for malachite green removal are virtually absent from literature. This gap highlights both the novelty and the scientific originality of the present work.

The aim of this study is to develop and evaluate novel bio-based materials derived from fish processing waste, specifically fish bladder (FB) and fish scales (FS), as low-cost, sustainable supports for adsorbents and/or catalysts. By valorizing underutilized fish waste and exploring its dual functionality in adsorption and catalytic ozonation, this research introduces a new direction in the field of water treatment technologies. The originality of the approach lies in the use of fish-derived biomaterials, rarely explored in this context, combined with vanadium functionalization to enhance oxidative degradation of organic pollutants. This dual strategy not only contributes to circular economy principles by repurposing biological waste, but also proposes a novel catalytic system with potential for scalable environmental applications.

## 2. Materials and Methods

Biomaterials such as FB and FS, along with their vanadium-impregnated counterparts (FB-V and FS-V), were obtained from biological waste sources from a certified fish retailer authorized for the sale of fish (*Cyprinus carpio*) products. Vanadium incorporation was achieved using vanadium (IV) oxide sulfate hydrate (VO-SO_4_·xH_2_O, 97%, supplied by Aldrich, Lot MKCM8324, manufactured in Tokyo, Japan). Distilled water, supplied by the laboratory purification system, from a GFL 2008 distillation unit (Burgwedel, Germany), was used throughout the preparation process.

### 2.1. Biomaterials Synthesis

The biomaterials FB, FB-V, FS, and FS-V were synthesized through a sequence of operations designed to preserve the structural integrity of the biological substrates and ensure reproducibility. The raw fish bladder and fish scales underwent thorough rinsing with distilled water to eliminate residual impurities, followed by drying in a ventilated oven at 40 °C for 10 h to prevent thermal degradation of proteinaceous components. The dried materials were then ground using a stainless-steel grinder and sieved to a particle size of 250 µm to ensure uniformity in texture and surface area, and the final particle size of the biosorbent materials was 250 ± 0.3 µm. Vanadium incorporations into FB or FS were performed via wet impregnation using an aqueous solution of vanadium (IV) oxide sulfate hydrate at a concentration of 1 mmol V per gram of support. For each 3 g of substrate, 20 mL of vanadium solution was added dropwise under continuous stirring to ensure complete wetting and optimal interaction with the support matrix. The impregnated samples were dried in a laboratory oven at 120 °C for 2 h. This impregnation–drying cycle was repeated three times, with intermediate cooling to room temperature, to promote uniform distribution and anchoring of vanadium species within the porous structure. Final materials were stored in desiccators to prevent moisture uptake and preserve their physicochemical properties prior to characterization and application.

### 2.2. Experimental Section

X-ray diffraction (XRD) analysis was carried out using a SmartLab diffractometer (Rigaku Corporation, Tokyo, Japan), operating in Bragg–Brentano geometry with a copper radiation source (λ = 1.5406 Å). Data acquisition was performed over a 2θ range from 2° to 90°. The step size was 0.02° and the scanning rate was 3° per minute.

Infrared spectra were recorded using an IRSpirit spectrometer (Shimadzu Corporation, Kyoto, Japan), equipped with a single-reflection ATR accessory featuring a diamond crystal and a DLATGS detector (DLATGS， Dresden, Germany). The measurements were carried out in transmittance mode, with a resolution of 8 cm^−1^, averaging 45 scans per minute over the spectral range of 400–5000 cm^−1^. This configuration enables direct analysis of solid and semi-solid samples without the need for extensive preparation.

The Verios G4 UC scanning electron microscope (Thermo Fisher Scientific, Waltham, MS, USA) was used to investigate the microstructural features and elemental composition of the samples. The device was coupled with an energy-dispersive X-ray spectroscopy (EDS) system featuring an Octane Elect Super SDD detector (Mahwah, NJ, USA). A thin platinum layer (6 nm) was applied using a Leica EM ACE200 sputter coater (Leica Microsystems, Wetzlar, Germany) to enhance surface conductivity and reduce charging effects during imaging. The backscattered electron mirror detector (MD) (Thermo Fisher Scientific, Waltham, MS, USA) was used with an accelerating voltage of 15 kV.

Thermal behavior of synthesized materials was analyzed using a TGA 5500 instrument (TA Instruments, New Castle, DE, USA) through thermogravimetric analysis (TGA). A sample of approximately 6.01 mg was placed in a platinum crucible and heated under a nitrogen flow (25 mL·min^−1^) at a constant rate of 10 °C·min^−1^, reaching a final temperature of 700 °C. Throughout the heating process, mass variations were continuously monitored.

Agilent 6500 Series Accurate-Mass Q-TOF LC/MS system (Agilent Technologies, Santa Clara, CA, USA) was employed to perform high-performance liquid chromatography with mass spectrometry detection (HPLC-MS), enabling precise molecular analysis. The samples were analyzed by HPLC (Agilent Technologies 1200 Series, Santa Clara, CA, USA) on a ProntoSIL C18 column (125 mm × 4 mm, 5 μm). The separation conditions were as follows: column temperature at 30 ◦C, injection volume of 10 µL, flow rate of 1 mL·min^−1^ (with a 1:9 splitter for ESI-MS), and izocratic elution. The mobile phase consisted of 40% solvent A (water with formic acid 0.1%) and 60% solvent B (acetonitrile). The total acquisition time was 15 min, followed by column equilibration for 5 min before the next injection. The identification of MG and its degradation products was achieved using a Q-ToF mass detector (MS).

The Q-ToF MS was operated under the following conditions: electrospray ionization in positive mode nitrogen drying gas flow rate of 8 L·min^−1^, drying gas temperature of 325 °C, nebulizer pressure of 15 psig, capillary voltage of 4000 V, and fragmentor voltage of 100 V. The analysis was performed over an *m*/*z* range of 80–1500. Data acquisition and processing were carried out using MassHunter Workstation software data acquisition for 6200/6500 Series, version B.07.00 (Agilent Technologies, Santa Clara, CA, USA).

The experiments conducted in this study were designed as batch tests, performed under controlled laboratory conditions. This approach allows for the evaluation of the material’s performance in terms of adsorption capacity and catalytic degradation efficiency. The batch system was chosen to ensure reproducibility and to facilitate the preliminary assessment of the fish-derived biomaterials. Future studies may explore the potential for scaling up and applying these materials in continuous systems such as column setups.

The calculation formula for both adsorption and ozonation yields was based on absorbance measurements, using the following expression:

(1) $$Yield\;(\%)=\left(\frac{A_{0}-A_{t}}{A_{0}}\right)\times 100ּ$$

where *A*_0_ is the initial absorbance (before the reaction), *A_t_* is the absorbance at time *t* (after a certain reaction time).

## 3. Results

### 3.1. Materials Characterization

#### 3.1.1. XRD Analysis

**Figure 2:**
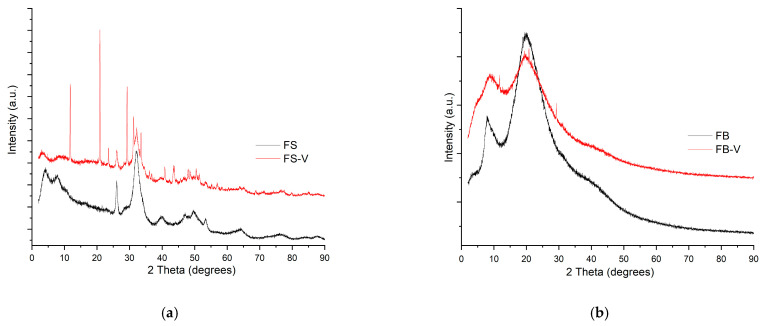
XRD diffractograms of vanadium-free and vanadium-impregnated fish-derived biomaterials: (**a**) FS and FS-V; (**b**) FB and FB-V.

The comparative analysis of the two XRD patterns, presented in Figure 2 reveals significant differences in the structural behavior of the FS/FS-V and FB/FB-V sample pairs following the applied treatment as evidenced by the presence of new additional crystalline peaks. In FS-V (Figure 2a), the new major peaks in FS-V at 11.79°, 23.55°, and 29.27° 2θ where assigned to the 011, 022 and 004 reflections in vanadium (IV) oxide sulfate hydrate (COD card no. 1527915). The diffraction peaks at 20.92°, 26.06° and 31.24° 2θ correspond to the 001, 201 and 400 reflections in vanadium (V) oxide (COD card no. 2020756). The rest of the additional less intense diffraction peaks can be traced to these two crystallographic phases. The presence of V_2_O_5_ in the sample indicates that following the treatment with vanadium (IV) oxide sulfate hydrate an oxidation process occurs upon the interaction with the substrate. In the FB-V pattern (Figure 2b), the new diffractions are barely distinguishable beneath the broad diffraction bands of the substrate. The two identified crystallographic phases are present in this sample as well as evidenced by the peaks at 11.79°, 23.55°, and 29.27° 2θ for vanadium (IV) oxide sulfate hydrate and 20.92°and 31.24° 2θ for vanadium (V) oxide, respectively.

Consequently, the two sets of samples demonstrate distinct structural responses to vanadium impregnation as evidenced by the presence of the specific diffraction peaks and the observation of the oxidation process that takes place following the impregnation.

#### 3.1.2. FTIR Analysis

**Figure 3:**
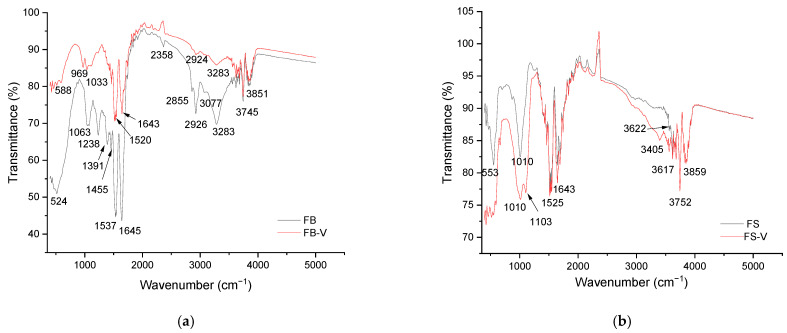
FTIR spectra of vanadium-free and vanadium-impregnated fish-derived biomaterials: (**a**) FB and FB-V; (**b**) FS and FS-V.

The FTIR spectra presented in Figure 3 compares the structural characteristics of various materials before and after vanadium incorporation. Across all samples, the presence of vanadium induces subtle or significant changes in the vibrational bands, indicating interactions with functional groups specific to each material type.

The FTIR spectra of the dried FB and its FB-V presented in Figure 3a show distinct changes that reflect vanadium interaction with protein structures. Variations in the regions associated with hydroxyl, amine, and aliphatic groups suggest chemical modifications following vanadium treatment. Shifts in vibrational peaks frequencies and relative changes in transmission spectra due to the absorbance of the Amide I and II bands suggest coordination between vanadium and protein carbonyl or amine groups, particularly near 1645 cm^−1^ (Amide I), 1525 cm^−1^ (Amide II), and 1063 cm^−1^ (Amide III), as well as in the N–H and O–H stretching regions. Additionally, the appearance of new bands below 1000 cm^−1^, attributed to V-O vibrations, confirms the incorporation of vanadium into the fish bladder matrix. Similar spectral features are observed in FS and FS-V presented in Figure 3b. The FS-V sample exhibits change that is consistent with vanadium binding to protein functional groups. As with FB-V, the emergence of bands below 1000 cm^−1^ supports the presence of vanadium-oxygen bonds, confirming successful integration of vanadium into the fish scale structure. Both FS and FS-V spectra display a band near 1010 cm^−1^, which is likely linked to the asymmetric stretching vibration v_3_(PO_4_^3−^), commonly found in phosphate-containing compounds like hydroxyapatite. Additionally, the sharp absorption peak at 1060–1110 cm^−1^ for both FS-V-FB-V are indicative of stretching vibrations associated with edge-sharing V–O and V=O bonds. This observation suggests the possible presence of phosphate groups within the fish scale-derived materials, likely originating from the biological composition of the tissue. Although FB and FB-V samples do not exhibit a similarly prominent peak in this region, the comparison with phosphate standards highlights structural differences between the two types of fish-derived biomaterials and supports the interpretation of functional group diversity. The absorption band observed in the 500–600 cm^−1^ region is attributed to calcium-oxygen bending vibrations, which may originate from the biological structure of the fish-derived materials, such as scales and swim bladder. Table 2 summarizes the FTIR band assignments for the fish bladder and fish scales, both untreated and vanadium treated. It presents the wavenumber ranges, associated functional groups, and corresponding interpretations for each spectral region.

The IR spectra confirm the presence and modification of functional groups associated with vanadium impregnation. These findings are consistent with EDAX analysis and elemental mapping, which clearly indicate the successful incorporation and distribution of vanadium within the material structure. The combined data support the functionalization process and its role in enhancing adsorption and catalytic performance. IR spectral changes observed in the study are consistent with previous findings. Matusoiu et al. investigated vanadium adsorption using a silica-iron oxide xerogel and confirmed, through IR analysis, the interaction between vanadium species and surface functional groups [41]. Similarly, Timoshchik et al. demonstrated that surface modification of amorphous silica with hydrazide groups enhances vanadium adsorption, with IR confirming complex formation between vanadium polyanions and functional groups [42]. These studies support the interpretation of our IR data and validate the efficiency of vanadium impregnation in fish-based materials.

#### 3.1.3. Scanning Electron Microscopy (SEM) Analysis

**Figure 4:**
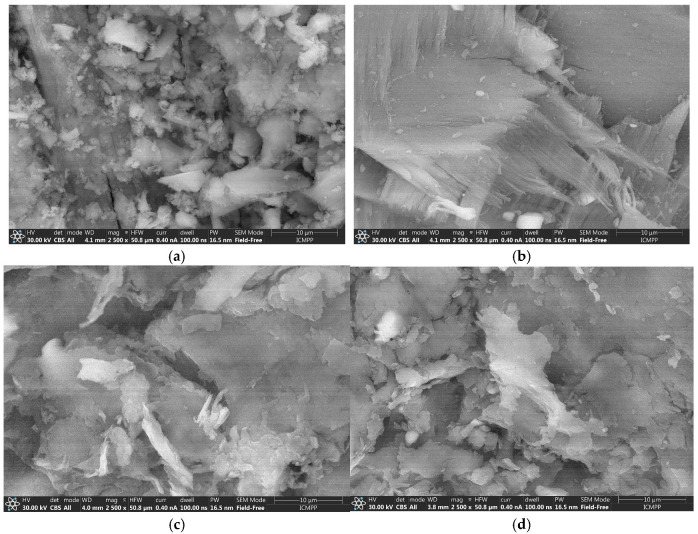
SEM analysis of: (**a**) FS; (**b**) FS-V; (**c**) FB; (**d**) FB-V.

Figure 4a, b, reveal distinct morphological differences between the FS and the FS-V. The untreated FS sample exhibited an irregular surface topology with heterogeneous particle sizes and natural textural features typical of biological materials. In contrast, the FS-V sample displayed a more uniform and granular surface, with visible crystalline structures likely corresponding to vanadium-based deposits. These morphological changes suggest successful surface modification through vanadium impregnation.

SEM images presented in Figure 4 revealed distinct morphological differences between raw and vanadium-functionalized fish-derived biomaterials. FS displayed a fibrous and porous structure, consistent with its collagen-hydroxyapatite composition, rich in calcium and phosphate groups. Upon vanadium treatment (FS-V), the surface became more compact and granular, suggesting the formation of vanadate species anchored to functional groups, enhancing surface reactivity.

Similarly, FB (fish bladder) exhibited a heterogeneous, porous morphology, favorable for adsorption. In contrast, FB-V showed a denser and more uniform surface with fine aggregates, indicating vanadium incorporation. These structural transformations support the hypothesis that vanadium-functionalized fish biomaterials present improved physicochemical properties, making them promising candidates for environmental applications such as bio-adsorption and catalysis.

#### 3.1.4. EDX Analysis

**Figure 5:**
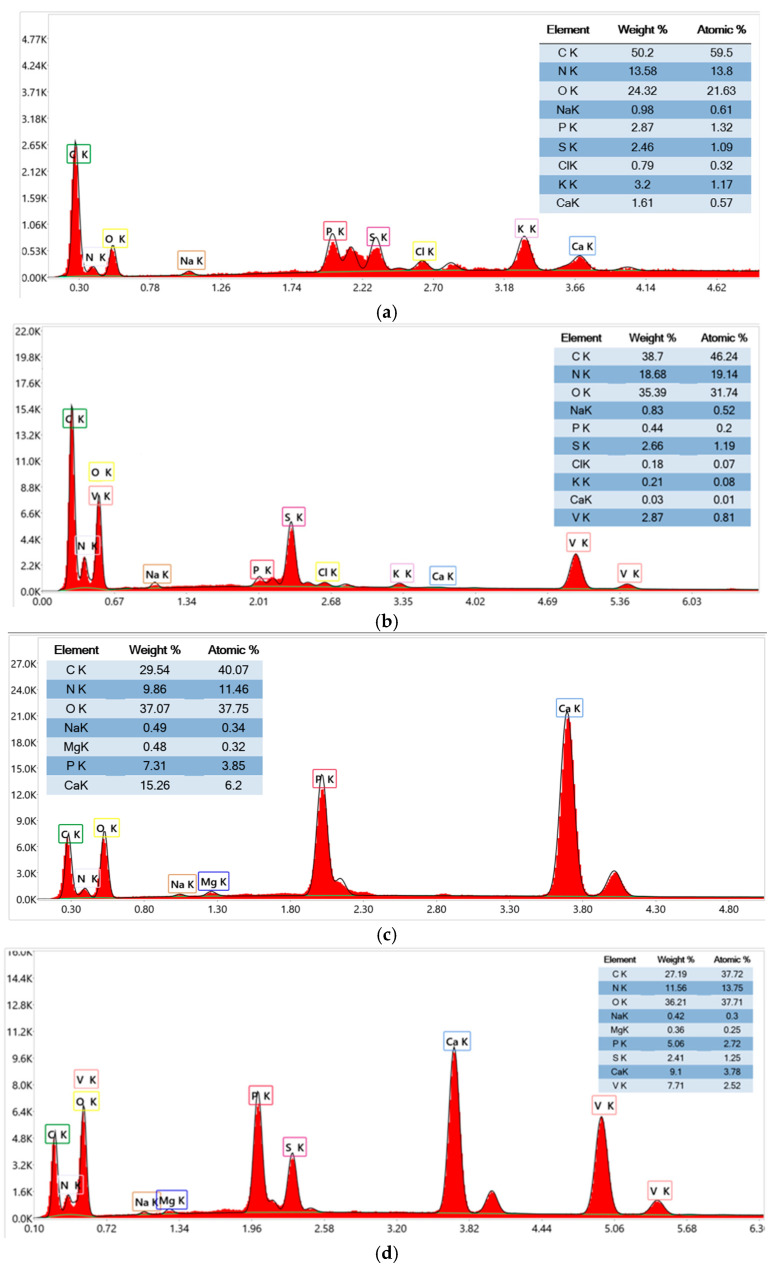
EDX analysis of (**a**) FB; (**b**) FB-V; (**c**) FS; (**d**) FS-V.

The EDX analysis comparing FB and FB-V, presented in Figure 5a,b reveals moderate vanadium incorporation (2.87 wt%) in FB-V. This is accompanied by an increase in oxygen (24.32 wt% to 35.39 wt%) and nitrogen (13.58 wt% to 18.68 wt%), suggesting partial coordination of vanadium with hydroxyl and amine groups. However, the relatively low vanadium content and the persistence of elements such as P, S, Cl, K, and Ca indicate limited chemical substitution and incomplete functionalization. These results suggest that the fish bladder matrix exhibits lower affinity or accessibility for vanadium compared to other biomaterials.

The EDX analysis reveals compositional changes between FS and FS-V, presented in Figure 5c,d, confirming chemical modification of the matrix. In FS-V, the carbon content slightly decreases from 29.54 wt% to 27.19 wt%, while oxygen remains nearly constant (37.07 wt% to 36.21 wt%), indicating preservation of the organic backbone. Nitrogen increases from 9.86 wt% to 11.56 wt%, suggesting enhanced interaction with nitrogen-containing groups, possibly due to vanadium coordination. Phosphorus and calcium decrease significantly (P: 7.31 wt% to 5.06 wt%; Ca: 15.26 wt% to 9.1 wt%), indicating partial leaching or substitution. Additionally, sulfur (2.41 wt%) and vanadium (7.71 wt%) are newly detected in FS-V, confirming successful impregnation. Minor variations in Na and Mg are observed, while atomic percentages follow similar trends. These changes support the incorporation of vanadium and functionalization of the fish scale matrix for potential catalytic or adsorptive applications. In comparison, FS-V shows significantly higher vanadium incorporation (7.71 wt%) than FB-V (2.87 wt%), along with more pronounced chemical changes in the matrix. These include reductions in phosphorus and calcium and the emergence of sulfur, indicating more effective functionalization. This suggests that fish scales (FS) have greater affinity and accessibility for vanadium than fish bladder (FB), making FS-V more suitable for catalytic or adsorptive applications.

**Figure 6:**
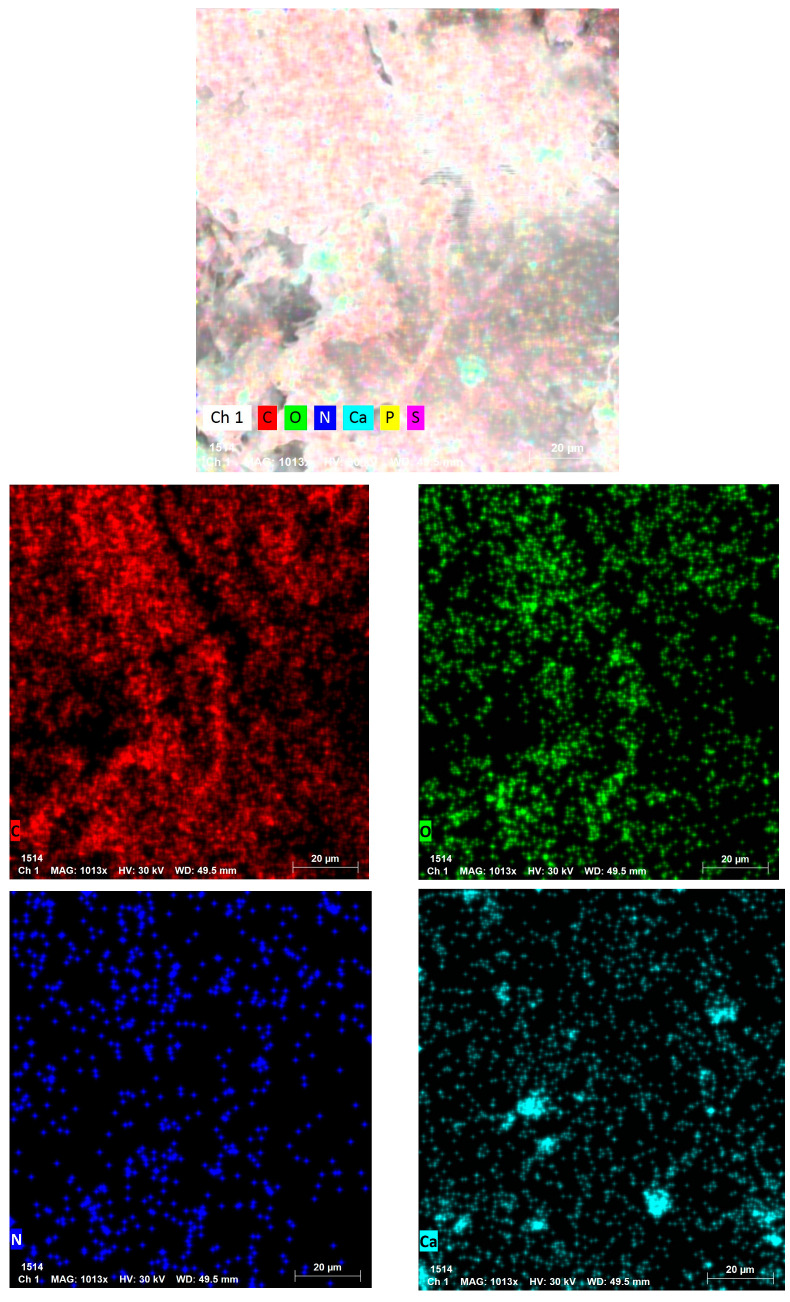

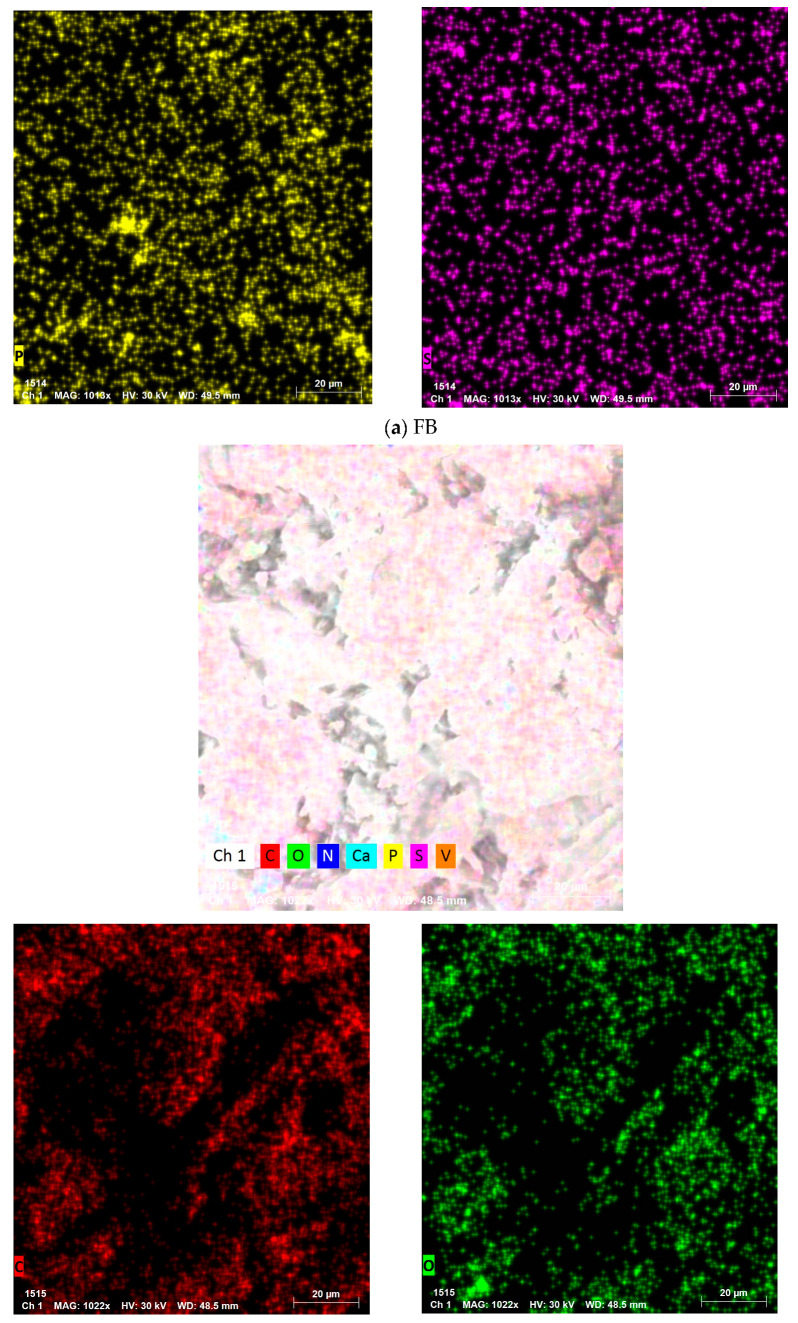

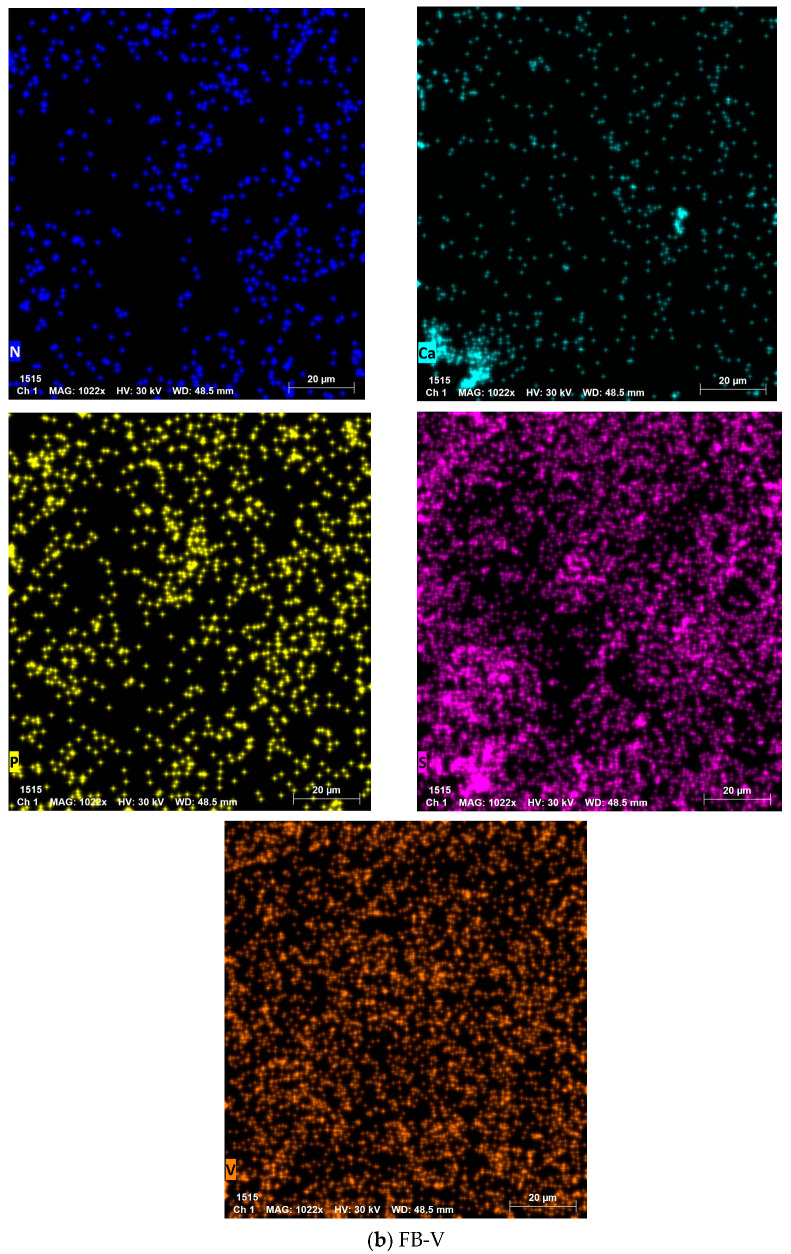

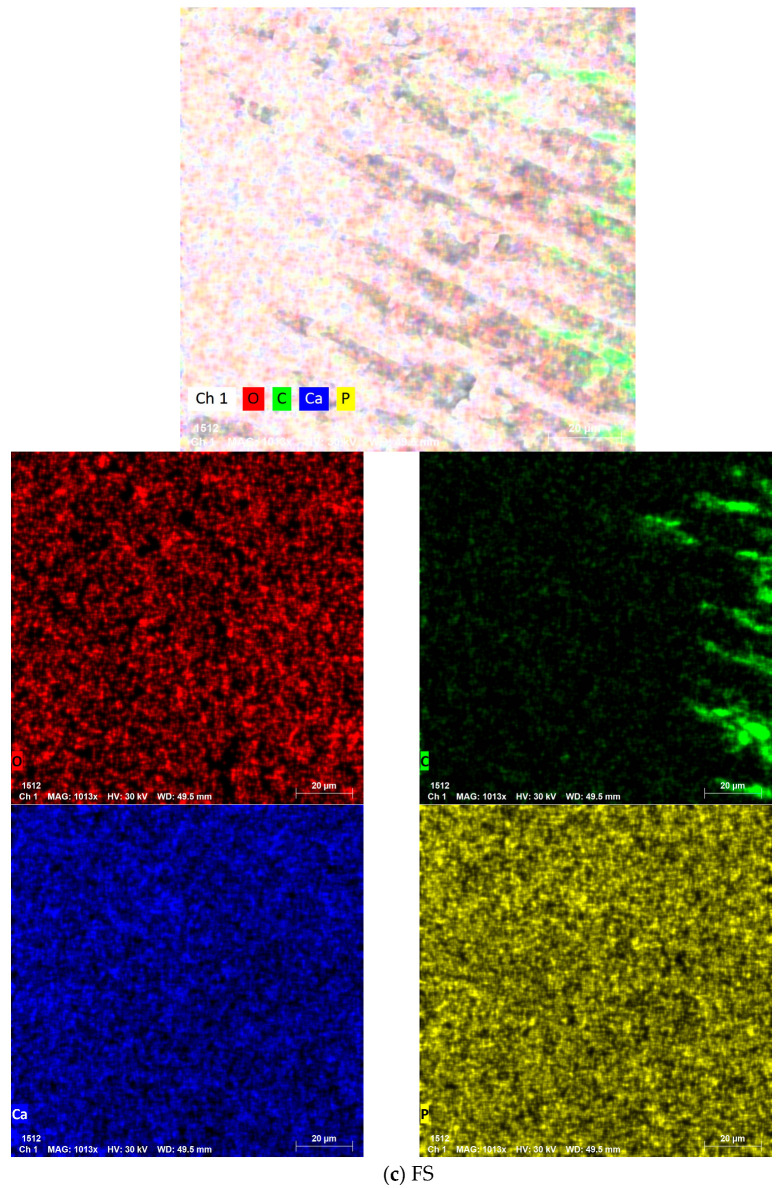

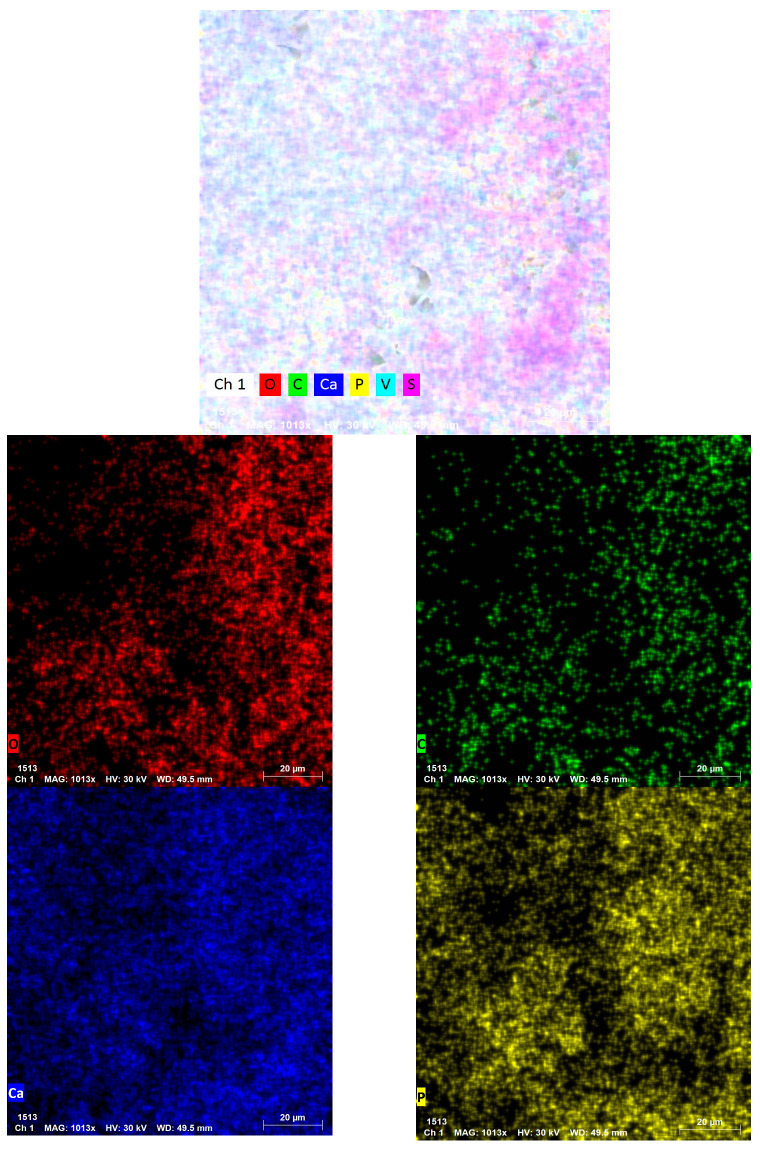

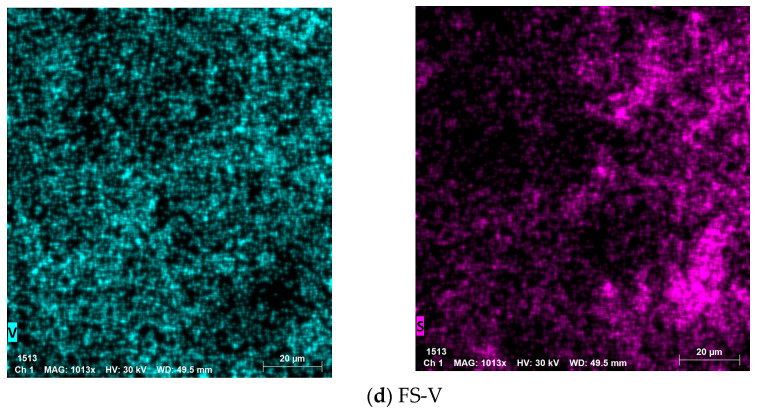
Elemental mapping of the tested biomaterials: (**a**) FB; (**b**) FB-V; (**c**) FS; (**d**) FS-V.

To highlight the homogeneous structure and elemental distribution within the tested biomaterials, elemental mapping characterization was performed (Figure 6). The mapping results support the uniform dispersion of the functional elements across the surface of the samples. These data reinforce the structural integrity and consistency of the materials used in the adsorption experiments.

Elemental mapping analysis revealed a uniform distribution of vanadium across both FS-V and FB-V samples. This homogeneous dispersion confirms the successful functionalization of the biomaterials and supports the structural consistency of the active sites involved in the adsorption and catalytic degradation of malachite green. Such uniformity is essential for ensuring reproducibility and efficiency in practical applications.

#### 3.1.5. TGA

**Figure 7:**
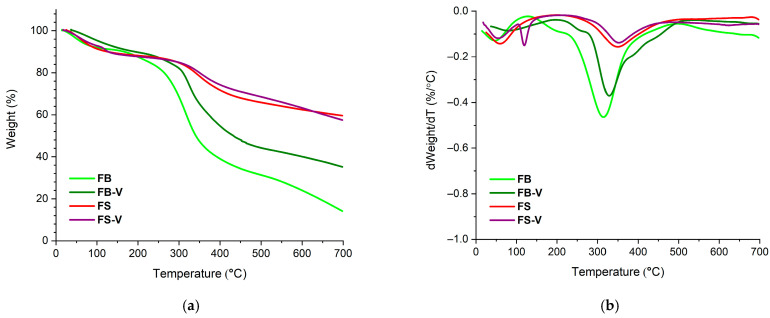
TGA (**a**) and DTG (**b**) analysis of: FB, FB-V, FS, FS-V.

The differences observed in the thermal degradation profile of fish scales and bladder (Figure 7) are correlated with their chemical composition.

FB are mainly composed of collagen and hydroxyproline, while FS have a more complex composition consisting of collagen (often less than bladder) (organic content), hydroxyapatite (anorganic content) and sometimes calcium carbonate and other proteins and glycoproteins. The thermal degradation of FB is dominated by collagen breakdown (200–500 °C); TGA curve drops sharply and leaves ~14% residue. FS have a higher onset decomposition temperature, decompose more slowly and leave ~60% residue due to hydroxyapatite content.

Peaks on the DTG curve correspond to temperatures where the mass loss rate is highest. FB shows sharper, more pronounced peaks at lower temperatures (315.35 °C) related to the organic phase decomposition, while FS presents some broader features related to both organic and inorganic degradation phases at higher temperatures (350.48 °C). The introduction of vanadium produces more important changes in the case of FB. The thermal stability of FB-V increases; the DTG peaks shifts from 315.35 °C to 328.34 °C, while the residue increases from 14% in the case of FB to 35% in the case of FB-V.

#### 3.1.6. HPLC-MS Analysis

**Figure 8:**
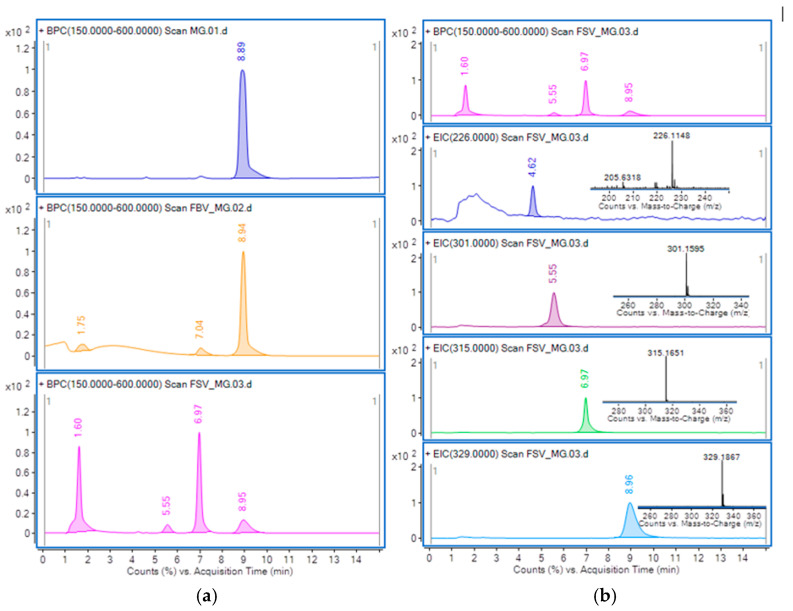
HPLC–MS profiles—(**a**) BPC of MG initial solution, MG and its intermediate products of degradation from the sample remained after catalytic ozonation in presence of FB-V and FS-V catalyst; (**b**) EIC chromatograms of MG and its intermediates along with the positive ESI-MS spectra of compounds identified in the case of FS-V catalyst.

The chromatographic separation of MG (5 × 10^−5^ M) and its intermediate products of degradation remaining the sample after catalytic ozonation, using FB-V and FS-V biomaterials as catalysts (60 mg, 0.5 g·h^−1^ O_3_, 30 min ozonation) are presented in Figure 8a. The MG and its intermediate products of degradation were separated on ProntoSIL C18 column, ionized in the positive electospray ionization (ESI+) mode and detected using Q-ToF analyzer. The base peak chromatogram (BPC) of the initial MG solution shows a single peak at retention time (*RT*) 8.9 min, corresponding to the singly charged ion [M]^+^ with *m*/*z* 329. In contrast, the BPC of the ozonated samples display multiple signals. Analysis of the BPC and EIC chromatograms revealed several MG degradation intermediates, including 4-(dimethylamino)phenyl-phenyl-methanone (*RT* 4.6 min, *m*/*z* 226.98), didemethyl-MG (*RT* 5.5 min, *m*/*z* 301.45), and desmethyl-MG (*RT* 6.9 min, *m*/*z* 315.45). These intermediates are consistent with compounds reported in previous studies [43].

Although only a qualitative assessment of MG and its degradation products was performed by HPLC–MS, the BPC of the samples subjected to catalytic ozonation (Figure 8a) clearly show that the signal corresponding to MG at 8.9 min is significantly reduced when using the FS-V catalyst, confirming its higher degradation efficiency compared to FB-V.

Overall, HPLC–MS allows precise monitoring of these intermediates, making it a powerful tool for investigating the degradation efficiency of MG under catalytic ozonation.

### 3.2. MG Adsorption

The experiments were conducted at an agitation speed of 350 rpm, which ensured effective mixing and contact between the biosorbent and the pollutant solution. All tests were performed at ambient temperature, reflecting typical environmental conditions and avoiding thermal interference with the adsorption or catalytic processes. The biosorbent-to-solution ratio was maintained at 10 mL of pollutant solution per experimental condition, with varying biosorbent doses depending on the specific test. These parameters were selected to ensure reproducibility and consistency across all experiments.

#### 3.2.1. Influence of Adsorbent Dosage

In the case of the influence of adsorbent dosage on the removal efficiency of the dye pollutant MG, the results obtained are presented in Table 3. These findings are consistent with the scientific literature, which indicates that increasing the amount of adsorbent generally enhances dye removal efficiency due to the greater availability of active surface area [28].

The experimental results presented in Table 3 demonstrate a clear correlation between adsorbent dosage and adsorption yield for both FS and FB biomaterials. As the dosage increases from 30 to 80 mg·L^−1^, the removal efficiency of MG dye improves significantly for both adsorbents. However, the performance and behavior of FS and FB differ notably.

FS exhibits high adsorption efficiency even at low dosages, starting at 83.60% and reaching a peak of 91.24% at 70 mg·L^−1^. The slight decrease at 80 mg·L^−1^ (89.77%) may suggest surface saturation or particle aggregation, which can reduce the effective surface area available for adsorption. This behavior aligns with findings in the literature, where adsorbent saturation at higher dosages can lead to overlapping of active sites and reduced dye uptake per unit mass [44]. In contrast, FB shows lower initial adsorption efficiency (33.28% at 30 mg·L^−1^), but a steep increase with dosage, reaching 82.00% at 80 mg·L^−1^. This suggests that FB requires a higher dosage to achieve comparable performance, likely due to lower surface area or fewer active functional groups. The gradual increase in efficiency is consistent with the principle that higher adsorbent mass provides more active sites for dye binding [45].

The observed trends are supported by adsorption theory, which states that increasing adsorbent dosage enhances the availability of active sites, leading to improved pollutant removal [46]. However, beyond a certain point, the efficiency may plateau or decline due to site saturation or interference between particles. Moreover, the adsorption of MG is typically governed by electrostatic interactions, π–π stacking, and hydrogen bonding between the dye molecules and functional groups on the adsorbent surface [46]. FS, being rich in hydroxyapatite and collagen, likely offers more favorable binding sites compared to FB, which may have a more compact or less reactive matrix.

FS proves to be a more efficient adsorbent for MG removal at lower dosages, indicating higher affinity and accessibility of active sites. FB, while capable of achieving similar efficiency, requires greater material input, which may affect its cost-effectiveness and scalability. These findings support the selection of FS for applications where rapid and efficient dye removal is essential.

These adsorption mechanisms are consistent with the chemical structure of MG, which contains aromatic rings and amine groups capable of forming π–π interactions and hydrogen bonds with the functional groups present on the biosorbent surface. The presence of hydroxyapatite and collagen in FS enhances these interactions, contributing to its higher affinity. Additionally, the incorporation of metal species may facilitate electron transfer processes during catalytic ozonation, supporting both adsorption and degradation pathways.

#### 3.2.2. Influence of Contact Time During Adsorption

**Figure 9:**
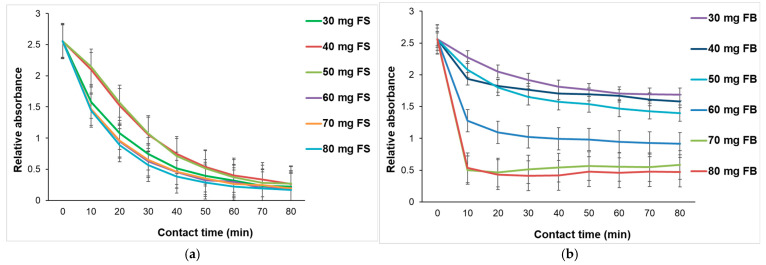
Effect of contact time on the adsorption of MG, at 618 nm using 10 mL of MG 5 × 10^−5^ M solutions and varying adsorbent dosages from 30 mg to 80 mg: (**a**) FS; (**b**) FB.

Figure 9a illustrates the kinetic profile of MG adsorption onto FS biomaterials at varying dosages. The data clearly show a rapid initial decrease in relative absorbance within the first 10–40 min, indicating a fast adsorption phase. This is followed by a plateau, suggesting that adsorption equilibrium is reached as the available active sites become saturated. This behavior is typical of pseudo-first-order or pseudo-second-order adsorption kinetics, where the initial phase is dominated by surface adsorption due to abundant active sites, and the later phase reflects slower intraparticle diffusion or equilibrium establishment [47]. The use of average curves across different dosages helps minimize experimental variability and provides a reliable representation of the overall adsorption behavior. This approach is supported by Shaikhiev et al. [48], who demonstrated that fish scales are effective biosorbents for dyes and that chemical modification or metal incorporation (e.g., vanadium) can significantly enhance their adsorption capacity. The plateau phase observed across all dosages also suggests that higher dosages do not drastically alter the equilibrium time, but rather increase the total adsorption capacity, as confirmed by the increasing adsorption yields from Table 3.

Figure 9b presents the adsorption kinetics of MG dye onto fish bladder (FB) biomaterial at various dosages (30–80 mg). The graph shows a rapid initial decrease in relative absorbance within the first few minutes of contact time, indicating a fast adsorption phase. This initial drop is typically conducted by external diffusion of dye molecules and the availability of active surface sites on the FB matrix.

Following this phase, the absorbance values gradually stabilize, suggesting that adsorption equilibrium is reached. The equilibrium phase reflects the saturation of available binding sites and the establishment of a dynamic balance between adsorbed and free dye molecules. As the dosage increases, the final absorbance values decrease, indicating improved adsorption efficiency at higher FB concentrations due to the greater availability of active sites. The presence of error bars across the curves confirms the reproducibility and consistency of the experimental data, supporting the reliability of the observed trends.

The increase in adsorption efficiency with higher FB dosages aligns with the principle that more adsorbent mass provides a greater number of active sites for dye binding. Although FB shows moderate adsorption capacity, its performance can be enhanced through chemical modification. Liang et al. [49] demonstrated that vanadium doping of magnetite (Fe_3−x_V_x_O_4_) significantly improves adsorption of dyes like methylene blue by increasing surface hydroxyl groups and enhancing electronic interactions. These mechanisms are applicable to FB-V, where vanadium likely contributes to higher surface reactivity and additional binding sites. Additionally, Avansi et al. [50] showed that vanadium pentoxide (V_2_O_5_) nanostructures exhibit strong adsorption for cationic dyes due to electrostatic interactions between dye molecules and the negatively charged adsorbent surface. While FB is not composed of V_2_O_5_, similar interactions may occur in FB-V due to the presence of vanadium species.

#### 3.2.3. Influence of pH Variation During Adsorption

**Figure 10:**
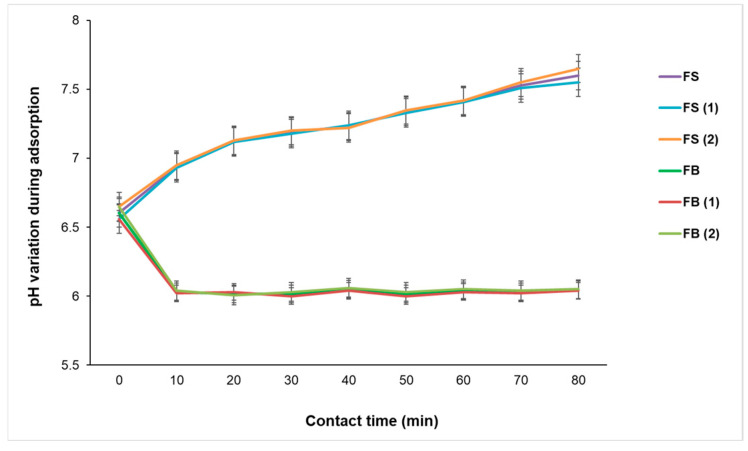
Variation in pH during MG adsorption on fish-derived biomaterials FS and FB.

The experimental data presented in Figure 10 reveals distinct pH evolution profiles for FS and FB biomaterials (60 mg) during the adsorption of MG. In the case of FS, the pH increases progressively from approximately 6.0 to 7.5 within the first 30–40 min of contact time, after which it stabilizes. This behavior suggests that the adsorption process involves proton exchange mechanisms, likely through the consumption of H^+^ ions or release of OH^−^ ions from the bio-sorbent surface. Such mechanisms are characteristic of materials containing hydroxyapatite, which is abundant in fish scales and known for its ion-exchange capacity and surface reactivity with cationic dyes. This observation is supported by Wei et al. [51] who demonstrated that hydroxyapatite and its derivatives possess active surface groups capable of participating in ion exchange and deprotonation reactions, which can influence the pH of the surrounding medium during adsorption processes. Additionally, Shaikhiev et al. [48] confirmed that fish scales, due to their mineral composition, particularly phosphate and calcium groups, exhibit pH-dependent adsorption behavior, with increased pH linked to interactions between dye molecules and surface functional groups.

In contrast, FB biomaterials maintain a relatively stable pH around 6.0 throughout the adsorption process. This suggests a less chemically reactive surface, where adsorption likely occurs through physical interactions or weak electrostatic forces, rather than through mechanisms that significantly alter the pH of the solution. This behavior is consistent with literature on collagen-based biosorbents, which typically exhibit minimal pH variation during dye adsorption. Azadi et al. [52] demonstrated that collagen-based cryogels used for cationic dye removal (e.g., Rhodamine B) operate effectively at neutral pH and rely primarily on electrostatic and hydrogen bonding interactions, without significant involvement of proton exchange.

### 3.3. Catalytic Ozonation

#### 3.3.1. Influence of the Catalyst Mass, Ozonation Time and O_3_ Dose

**Figure 11:**
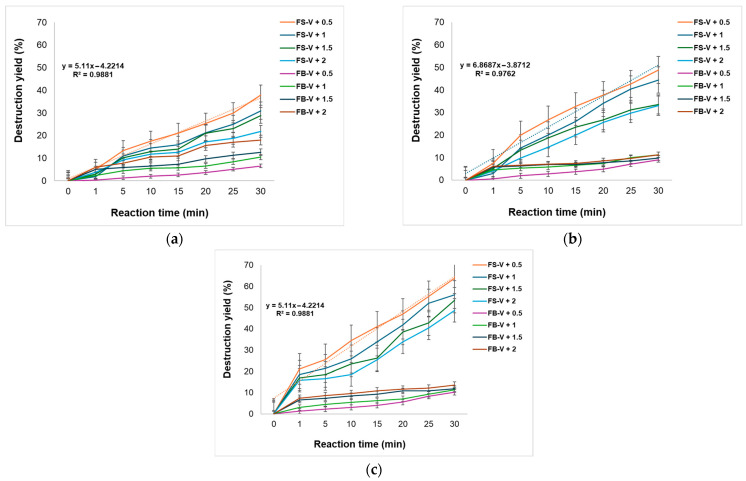
Catalytic degradation of MG monitored at 618 nm using (**a**) 40 mg; (**b**) 60 mg; and (**c**) 80 mg of FS-V or FB-V catalysts under 0.5, 1, 1.5 and 2 g O_3_·h^−1^, used for 10 mL MG 5 × 10^−5^ M.

Figure 11 illustrates the catalytic degradation of MG at 618 nm using 40, 60 and 80 mg of FS-V or FB-V, under ozone flow rates of 0.5, 1, 1.5, and 2 g O_3_·h^−1^. The experiments were performed in duplicate, and error bars indicate standard deviation.

The graph analyzed in Figure 11a presents the catalytic degradation of MG at 618 nm, using 40 mg of FS-V or FB-V catalysts under four different ozone flow rates (0.5, 1, 1.5, and 2 g O_3_·h^−1^). The results highlight a distinct evolution between the two catalysts depending on the intensity of the ozone flow. In Figure 11b, for a dosage of 60 mg, a general increase in degradation efficiency is observed compared to the 40 mg case, suggesting a more effective activation of the catalytic surface. FS-V remains superior to FB-V under all tested conditions, although the differences slightly decrease, indicating a possible saturation of catalytic efficiency at higher dosages. At a dosage of 80 mg, shown in Figure 11c, the FS-V catalyst achieves the highest degradation efficiencies of MG observed in this study, particularly at lower ozone flow rates. This behavior indicates optimal catalytic activation, with a well-utilized active surface and an efficient distribution of vanadium species.

#### 3.3.2. Influence of pH During Catalytic Ozonation

**Figure 12:**
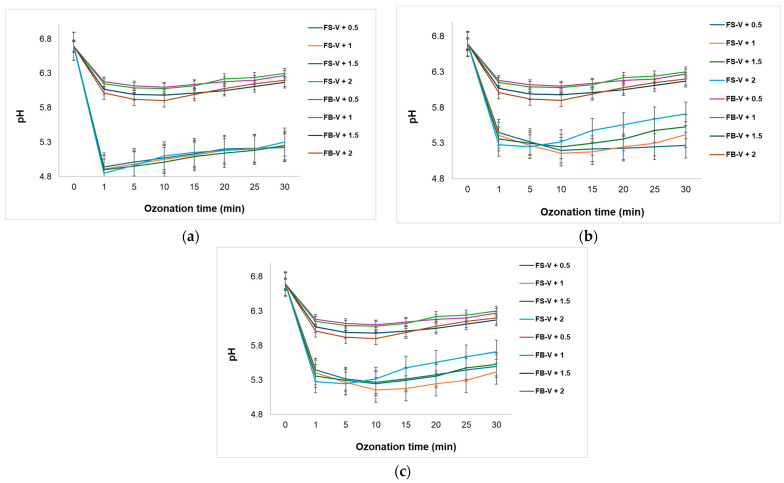
pH variation during ozonation using (**a**) 40 mg; (**b**) 60 mg; and (**c**) 80 mg of FS-V and FB-V catalysts under 0.5, 1, 1.5 and 2 g O_3_·h^−1^, 10 mL MG 5 × 10^−5^ M.

The evolution of pH during the catalytic ozonation process provides valuable insights into the reaction environment and the activity of the FS-V and FB-V catalysts under varying ozone flow rates and catalyst dosages, as illustrated in Figure 12.

In Figure 12a, for a dose of 40 mg of FS-V or FB-V catalysts, a sharp initial drop in pH is observed within the first few minutes, more pronounced at higher ozone flow rates (1.5 and 2 g O_3_·h^−1^). This rapid acidification suggests the formation of acidic compounds, such as carboxylic acids or aldehydes, resulting from the partial oxidation of MG dye. In Figure 12b, the pH behavior during catalytic ozonation with 60 mg of FS-V and FB-V is characterized by an initial drop followed by a gradual increase, aligning with findings reported by Mello et al. [53], who demonstrated that natural biocatalysts such as pyrolusite can promote pH stabilization and recovery due to their buffering capacity and controlled interaction with ozone. In Figure 12c, the pH evolution observed with 80 mg of FS-V and FB-V catalysts reveals a distinct pattern: a sharper initial drop in pH within the first minute of ozonation, followed by a more consistent and gradual increase over the remaining 25–30 min. The final pH values at 30 min are higher than those observed at lower dosages, suggesting that the increased amount of biomaterial enhances the system’s ability to neutralize acidic intermediates.

## 4. Discussion

Fish-derived biomaterials such as scales and bladders are protein-rich matrices with distinct structural and functional characteristics that influence their catalytic behavior. Fish scales are composed of a dual-layer structure: a bony layer containing hydroxyapatite and collagen, and a fibrous layer formed by interwoven collagen fibers [54]. This architecture provides both mechanical strength and a high surface area, which are advantageous for catalyst loading and interaction with reactants. In contrast, the fish bladder is primarily made up of collagen and elastin, forming a flexible and porous structure with moderate surface area. Although less rigid than scales, the bladder’s protein matrix still offers functional groups capable of interacting with metal ions. Both materials contain collagen-based functional groups such as amino (-NH_2_) and carboxyl (-COOH) moieties, which can effectively chelate vanadium ions, stabilizing them within the matrix and enhancing their catalytic activity. These interactions facilitate the generation of reactive oxygen species (ROS) during ozonation, contributing to the degradation of organic pollutants like MG. Moreover, the PI3K-AKT signaling pathway, known to regulate fish scale development, influences the expression of structural proteins including col1a1, col6a2, krt8, and krt18. These proteins may play a role in determining the adsorption capacity and reactivity of the biomaterial, further supporting its function as a bio-catalyst [54].

The results obtained from the adsorption study confirm the direct influence of the adsorbent dose on the removal efficiency of MG, with superior performance for FS compared to FB. FS achieves high yields (83.60% ± 0.80) even at low doses (30 mg·L^−1^), indicating a well-exposed active surface and increased affinity for dye molecules. The slight decrease (91.24% ± 0.84, 89.77% ± 0.80) at higher doses (70, 80 mg·L^−1^) suggests saturation of active sites, a phenomenon well-documented in the scientific literature. FB requires higher doses (80 mg·L^−1^) to achieve comparable efficiencies, which may be attributed to a more compact surface or a lower number of functional groups. The progressive increase in efficiency with dose aligns with adsorption theory, which emphasizes the role of active site availability. From a kinetic perspective, both materials exhibit a rapid adsorption phase followed by equilibrium, behavior characteristic of pseudo-first or pseudo-second order models. FS favors ion exchange, reflected in the increase in pH, while FB maintains a stable pH, indicating lower reactivity. In conclusion, FS proves to be more efficient and cost-effective for rapid treatment of colored wastewater, while FB could be improved through chemical modifications to enhance its adsorption performance.

The short contact time (~30 min) required to reach adsorption equilibrium represents a practical advantage for real-world applications. Rapid adsorption kinetics are essential for scalable water treatment processes, where efficiency and operational speed are critical. This feature enhances the applicability of the biosorbent in continuous or batch systems.

The results obtained from the catalytic ozonation study revealed that, for the FS-V catalyst, a decrease in degradation efficiency was observed with increasing ozone flow rate: from approximately 38% at 0.5 g O_3_·h^−1^ to 28% at 2 g O_3_·h^−1^. This counterintuitive trend may be attributed to surface saturation of the catalyst or to the excessive formation of reactive species (such as ozonides or peroxides), which can inhibit the main degradation reaction. Under conditions of ozone excess, hydroxyl radicals may be consumed in secondary reactions, leading to a reduction in the overall process efficiency. This phenomenon is known in the scientific literature as “inhibition at high oxidant concentrations”, and has been reported in studies on heterogeneous catalytic ozonation [14].

In contrast, the FB-V catalyst exhibits an opposite trend: the degradation efficiency increases with the intensification of the ozone flow rate, from approximately 6.5% at 0.5 g O_3_·h^−1^ to 18% at 2 g O_3_·h^−1^. This behavior suggests that FB-V undergoes more effective activation in the presence of excess ozone, possibly due to a less reactive surface, which requires a higher oxidant concentration to generate the radicals necessary for degradation. Thus, FB-V appears to benefit from increased ozone flow to compensate for its intrinsically lower catalytic activity, which aligns with observations in the literature regarding catalysts with low activity under standard conditions that become activated in the presence of excess oxidant [55]. This divergence between FS-V and FB-V highlights the importance of optimizing the ozone flow rate according to the nature of the catalyst. FS-V is more efficient at lower ozone flow rates, but its performance declines at higher concentrations, whereas FB-V becomes more active as the ozone flow increases. These observations are essential for the design of treatment processes for MG-contaminated water, indicating that the choice of catalyst must be correlated with the applied oxidation conditions.

For the FS-V catalyst, the degradation efficiencies obtained at a dosage of 60 mg range from approximately 48% at 0.5 g O_3_·h^−1^ to 33% at 2 g O_3_·h^−1^, confirm the decreasing trend previously observed. This decline can be attributed to the excessive formation of free radicals, which may participate in secondary reactions or inhibit the main degradation pathway. The phenomenon is well documented in scientific literature, as described by Li et al. [14], who demonstrated that excessive ozone flow can reduce mineralization efficiency in heterogeneous catalytic ozonation processes.

In the case of the FB-V catalyst, degradation efficiencies increase from approximately 9% at 0.5 g O_3_·h^−1^ to 11% at 2 g O_3_·h^−1^, confirming the behavior observed at the 40 mg dosage. This result suggests that FB-V exhibits activation dependent on ozone concentration, being more effective under intense oxidation conditions. This type of behavior is supported by the study conducted by Mostafa and Amdeha [56], who demonstrated that heterogeneous structures based on ZnVFeO_4_ show enhanced activation under visible light and in the presence of hydroxyl radicals generated by ozone. Moreover, recent studies on vanadium- and iron-based catalysts indicate that increasing the catalyst dosage can improve process efficiency up to a certain threshold, beyond which saturation or inhibition phenomena may occur. For example, in the study by Khezami et al. [57], the degradation of MG using vanadium-doped ZnO followed first-order kinetics, but the efficiency was negatively affected at high oxidant concentrations. Thus, at a dosage of 60 mg, FS-V remains more efficient than FB-V, but its efficiency decreases with increasing ozone flow, confirming the existence of an optimal oxidant threshold for this material.

In contrast, FB-V benefits from higher ozone flow rates, indicating a positive dependence on the oxidant and progressive activation under intense oxidation conditions. These results are consistent with scientific literature and emphasize the importance of optimizing both catalyst dosage and ozone flow according to the nature of the material used. The correct selection of these parameters is essential for maximizing the efficiency of water treatment processes, especially in the case of pollutants such as MG. The degradation efficiencies obtained with FS-V at 80 mg increase significantly during the first 30 min of reaction, reaching values of 63.84% at 0.5 g O_3_·h^−1^, 56.10% at 1 g O_3_·h^−1^, 53.51% at 1.5 g O_3_·h^−1^, and 48.67% at 2 g O_3_·h^−1^. A decreasing trend in efficiency is again observed with increasing ozone flow, confirming the hypothesis that excess oxidant may lead to the formation of unstable free radicals or secondary reactions that inhibit the main degradation process. This phenomenon is well documented in the scientific literature, including the study by Li et al. [14], which emphasizes that mineralization efficiency can be negatively affected at high ozone concentrations.

In the case of the FB-V catalyst, the degradation efficiencies are significantly lower, but show a steady increase with the intensification of the ozone flow. The values at 30 min are: 10.20% at 0.5 g O_3_·h^−1^, 11.36% at 1 g O_3_·h^−1^, 11.84% at 1.5 g O_3_·h^−1^, and 13.65% at 2 g O_3_·h^−1^. This behavior suggests that FB-V activation is dependent on ozone concentration, being more effective under intense oxidation conditions. This type of activation is supported by the study conducted by Mostafa and Amdeha [56], who demonstrated that heterogeneous structures based on ZnVFeO_4_ exhibit enhanced activation in the presence of hydroxyl radicals generated by ozone.

In conclusion, at a dosage of 80 mg, FS-V confirms its superiority in the degradation of MG, achieving maximum efficiencies at moderate ozone flow rates. However, its efficiency slightly decreases at higher flow rates, indicating the presence of an optimal ozone concentration threshold. FB-V remains less efficient, but responds positively to increased ozone flow, making it suitable for applications where intense oxidation is required. These results reinforce the observations made at 40 and 60 mg, and highlight the importance of adapting reaction conditions to the nature of the catalyst.

The variation in pH during catalytic ozonation is a key indicator of the chemical processes occurring in the solution, particularly the formation of intermediate species and the efficiency of organic pollutant mineralization. The FS-V and FB-V catalysts exhibit slightly different behaviors, indicating that the structure and composition of the biomaterial influence the ozonation mechanism. Notably, FS-V appears to induce a faster pH drop (from 6.69 to 4.85) at higher ozone flow rates, which may be attributed to more intense catalytic activity or a lower buffering capacity.

After reaching minimum pH values around minute five, both catalysts show a gradual increase in pH over the remaining ozonation time. These results are consistent with the study conducted by Aguilar-Rosero et al. [58], which emphasizes that pH directly influences the efficiency of bioadsorbents in water treatment processes. In particular, the decrease in pH is associated with the formation of oxidized compounds and the activation of adsorption sites on the surface of the biomaterial. On the other hand, the study by Salehi et al. [59], on enzymatic biocatalysts shows a stable pH during the oxidation of phenolic compounds, due to a controlled radical generation mechanism. This behavior differs from that observed in our experiment, where the FS-V and FB-V biomaterials do not maintain a constant pH but rather promote the accumulation of acidic species. Nonetheless, the observed pH recovery in the later stages of ozonation suggests that these biomaterials may also facilitate the neutralization or further oxidation of acidic intermediates, contributing to a more balanced reaction environment.

Therefore, the results obtained are aligned with studies on bioadsorbents but differ from those on enzymatic biocatalysts, confirming the distinct nature of the catalytic ozonation mechanism in the presence of vanadium-impregnated biomaterials. This difference can be attributed to the physicochemical structure of the biomaterial and its interaction with ozone, which favors the formation of oxidizing species and a biphasic pH behavior, initial acidification followed by partial recovery. The contrast between the findings in Figure 12b and those reported by Li et al. [60], who observed a continuous pH decline during ozonation with γ-Al_2_O_3_-based biocatalysts, highlights the importance of the biocatalyst’s origin and structure in modulating the ozonation pathway. In our case, the buffering and recovery effects observed with FS-V and FB-V suggest a more dynamic and adaptable reaction environment. Finally, the behavior observed at 80 mg reinforces the idea that higher biomaterial concentrations not only improve the buffering capacity, but also contribute to a more uniform and controlled ozonation environment, which is beneficial for achieving advanced mineralization and reducing the formation of persistent acidic by-products.

Future research should aim to deepen the understanding of the catalytic ozonation mechanism by investigating the molecular pathways of radical formation and intermediate species. Enhancing the surface properties of FS-V and FB-V through structural modifications could improve their catalytic performance and selectivity. Additionally, testing these biomaterials in real wastewater conditions is essential to evaluate their robustness and practical applicability. Long-term stability and regeneration studies are needed to assess their reusability, while integrating catalytic ozonation with other advanced oxidation processes may offer synergistic benefits for pollutant removal. Preliminary adsorption–desorption tests were performed in three cycles, but further detailed studies are needed to confirm the biosorbents’ long-term reusability. These directions will support the development of more efficient and sustainable water treatment technologies.

Also, future research directions will focus on expanding the characterization of the composite material beyond the optical studies already performed. UV-Vis spectroscopy results, presented in this manuscript, demonstrate the material’s ability to adsorb the organic dye MG, providing a solid foundation for evaluating the efficiency of the adsorption process. To further elucidate the catalytic behavior of the system, electrochemical investigations, such as cyclic voltammetry and electrochemical impedance spectroscopy, are planned. These techniques will enable a deeper understanding of the redox properties of the material and the catalytic role of vanadium in the degradation of MG. Additionally, a plausible reaction mechanism will be proposed, supported by schematic representations that integrate both optical and electrochemical data into a coherent model of the treatment process.

## 5. Conclusions

This study demonstrates the potential of fish-derived biomaterials (FS, FS-V, FB and FB-V) as sustainable supports for adsorption and catalytic ozonation in water treatment. FS-V showed superior performance in Malachite Green degradation, especially at 80 mg dosage and moderate ozone flow. The lower efficiency of FB-V under all tested conditions indicates limitations in its catalytic activity, requiring further investigation into its structure and activation mechanisms.

The study presents promising results regarding the adsorption and potential catalytic degradation of malachite green using the functionalized biomaterial. However, certain challenges remain, particularly the lower efficiency observed for the FB-V sample. To address these limitations and gain deeper insight into the material’s behavior, future work will focus on electrochemical investigations. These will complement the optical data already obtained and contribute to the development of a plausible reaction mechanism. This mechanism will be supported by schematic representations, integrating both optical and electrochemical findings into a coherent model of the treatment process.

Future research should focus on improving FB-V functionality, exploring other metal incorporations, and optimizing operational parameters to enhance overall treatment efficiency.

The use of fishery waste materials such as fish scales and swim bladders contributes to lowering the production cost of the biosorbents and biocatalysts, supporting the development of economically feasible treatment solutions.

## Figures and Tables

**Table 2:** FTIR band assignments.

Wavenumber (cm^−1^)	Functional Group/Vibration	Observed in Samples	Interpretation/Compound Type
~3400	O–H/N–H stretching	All samples	Hydroxyl groups, amines, adsorbed water
~3000–2800	C–H stretching (aliphatic)	All samples	Protein/lipid side chains
~1700–1600	Amide I (C=O stretching)	All samples	Protein backbone, carbonyl groups
~1600–1500	Amide II (N–H bending, C–N stretch)	All samples	Protein matrix, amine interactions
~1200–1000	C–O, C–N, P–O stretching	All samples	Polysaccharides, phosphates
~960–1000	V–O stretching	B-V, FS-V	Vanadium-oxygen bonds (vanadium incorporation)
~500–600	Ca–O stretching	All samples	Bending vibrations in the PO_4_ group

**Table 3:** Adsorption efficiency of MG dye on raw biomaterials.

Adsorbent Type	Adsorbent Dosage (mg·L^−1^)	Contact Time (min)	Adsorption Yield (η, %)
			MG 5 ×10^−5^ M
FS	30	60	83.60 ± 0.80
	40		84.19 ± 0.82
	50		85.52 ± 0.84
	60		88.20 ± 0.82
	70		91.24 ± 0.84
	80		89.77 ± 0.80
FB	30	60	33.28 ± 0.31
	40		34.68 ± 0.31
	50		42.66 ± 0.39
	60		62.94 ± 0.60
	70		78.36 ± 0.80
	80		82.00 ± 0.79

## Data Availability

The original contributions presented in this study are included in the article. Further inquiries can be directed to the corresponding authors.

## References

[1] Deng H., Zhang J., Huang R., Wang W., Meng M., Hu L., Gan W. (2022). Adsorption of malachite green and Pb^2+^ by KMnO_4_-modified biochar: Insights and mechanisms. Sustainability.

[2] Luan J., Hao L., Yao Y., Wang Y., Yang G., Li J. (2023). Preparation and Property Characterization of Sm_2_EuSbO_7_/ZnBiSbO_5_ Heterojunction Photocatalyst for Photodegradation of Parathion Methyl under Visible Light Irradiation. Molecules.

[3] El-Sayed N.M., Hikal W.M. (2015). Several staining techniques to enhance the visibility of Acanthamoeba cysts. Parasitol. Res..

[4] Culp S.J., Beland F.A. (1996). Malachite Green: A Toxicological Review. J. Am. Coll. Toxicol..

[5] Mirilă D.-C., Boudissa F., Beltrao-Nuñes A.-P., Platon N., Didi M.-A., Nistor I.-D., Roy R., Azzouz A. (2020). Organic Dye Ozonation Catalyzed by Chemically Modified Montmorillonite K10– Role of Surface Basicity and Hydrophilic Character. Ozone Sci. Eng..

[6] Hameed B., El-Khaiary M. (2008). Batch removal of malachite green from aqueous solutions by adsorption on oil palm trunk fibre: Equilibrium isotherms and kinetic studies. J. Hazard. Mater..

[7] Papinutti L., Mouso N., Forchiassin F. (2006). Removal and degradation of the fungicide dye malachite green from aqueous solution using the system wheat bran–Fomes sclerodermeus. Enzym. Microb. Technol..

[8] Hussain Hakami A.A., Ahmed M.A., Khan M.A., AlOthman Z.A., Rafatullah M., Islam M.A., Siddiqui M.R. (2021). Quantitative Analysis of Malachite Green in Environmental Samples Using Liquid Chromatography-Mass Spectrometry. Water.

[9] Asheghmoalla M., Mehrvar M. (2024). Integrated and hybrid processes for the treatment of actual wastewaters containing micropollutants: A review on recent advances. Processes.

[10] Dutta S., Gupta B., Srivastava S.K., Gupta A.K. (2021). Recent advances on the removal of dyes from wastewater using various adsorbents: A critical review. Mater. Adv..

[11] Rajabi M., Keihankhadiv S., Suhas, Tyagi I., Karri R.R., Chaudhary M., Mubarak N.M., Chaudhary S., Kumar P., Singh P. (2023). Comparison and interpretation of isotherm models for the adsorption of dyes, proteins, antibiotics, pesticides and heavy metal ions on different nanomaterials and non-nano materials—A comprehensive review. J. Nanostruct. Chem..

[12] Coppola D., Lauritano C., Palma Esposito F., Riccio G., Rizzo C., de Pascale D. (2021). Fish Waste: From Problem to Valuable Resource. Mar. Drugs.

[13] Rashed M.N., Gad A.A.E., Fathy N.M. (2024). Efficiency of chemically activated raw and calcined waste fish bone for adsorption of Cd (II) and Pb (II) from polluted water. Biomass Convers. Biorefin..

[14] Li X., Fu L., Chen F., Zhao S., Zhu J., Yin C. (2023). Application of Heterogeneous Catalytic Ozonation in Wastewater Treatment: An Overview. Catalysts.

[15] Rame R., Purwanto P., Sudarno S. (2023). A comprehensive review on catalytic ozonation: Emerging trends and future perspectives. Desalination Water Treat..

[16] Zhu H., Zou H. (2022). Ultra-efficient catalytic degradation of malachite green dye wastewater by KMnO_4_-modified biochar (Mn/SRBC). RSC Adv..

[17] Moumen A., Belhocine Y., Sbei N., Rahali S., Ali F.A.M., Mechati F., Hamdaoui F., Seydou M. (2022). Removal of Malachite Green Dye from Aqueous Solution by Catalytic Wet Oxidation Technique Using Ni/Kaolin as Catalyst. Molecules.

[18] Bilińska M., Bilińska L., Fronczak M., Kędzierska-Sar A., Kierzkowska-Pawlak H., Gmurek M. (2025). Application of metal oxides thin-film catalysts in structured catalytic ozonation reactor for dye and by-product detoxification. Sci. Rep..

[19] Liu Y., Chen C., Duan X., Wang S., Wang Y. (2021). Carbocatalytic ozonation toward advanced water purification. J. Mater. Chem. A.

[20] Shukla B.K., Sharma P.K., Goel A. (2024). Analysis of Chemical-Biological and Physical-Biological Hybrid Systems for Wastewater Treatment Utilizing Aeration and Ozonation. Lett. Appl. NanoBioSci..

[21] Szymański K., Mozia S., Ayral A., Brosillon S., Mendret J. (2023). Hybrid system coupling ozonation and nanofiltration with functionalized catalytic ceramic membrane for ibuprofen removal. Environ. Sci. Pollut. Res..

[22] Fu J., Liang F., Zhong W., Kuang T., Yin Z., Li Y., Huang Z., Liu H., Ma D. (2025). Enhanced catalytic degradation activity through quenching introduces Pd doping in TiO_2_ derived from NH_2_-MIL-125(Ti). Environ. Res..

[23] Zhao Z.-C., Wang K., Chang L., Yan R.-Q., Zhang J., Zhang M., Wang L., Chen W., Huang G.-B. (2023). Construction of S-scheme MIL-101(Fe)/Bi_2_MoO_6_ heterostructures for enhanced catalytic activities towards tetracycline hydrochloride photodegradation and nitrogen photofixation. Sol. Energy.

[24] Yang H., Zhao Z.-C., Yang Y.-P., Zhang Z., Chen W., Yan R.-Q., Jin Y., Zhang J. (2022). Defective WO_3_ nanoplates controllably decorated with MIL-101(Fe) nanoparticles to efficiently remove tetracycline hydrochloride by S-scheme mechanism. Sep. Purif. Technol..

[25] Hu E., Shang S., Chiu K.-L. (2019). Removal of Reactive Dyes in Textile Effluents by Catalytic Ozonation Pursuing on-Site Effluent Recycling. Molecules.

[26] Bilińska M., Bilińska L., Gmurek M. (2023). Homogeneous and Heterogeneous Catalytic Ozonation of Textile Wastewater: Application and Mechanism. Catalysts.

[27] Ali Z., Ikhlaq A., Qazi U.Y., Akram A., Ul-Hasan I., Alazmi A., Qi F., Javaid R. (2023). Removal of Disperse Yellow-42 Dye by Catalytic Ozonation Using Iron and Manganese-Loaded Zeolites. Water.

[28] Teweldebrihan M.D., Gnaro M.A., Dinka M.O. (2025). Adsorptive removal of malachite green dye from aqueous solution using Cordia africana leaf as biosorbent. Model. Earth Syst. Environ..

[29] Gul S., Afsar S., Shah T.A., Gul H., Aziz T., Zahra N., Alhomrani M., Alsanie W.F., Alamri A.S. (2025). White clover components as an effective biosorbent for the elimination of toxic malachite green from wastewater. Biomass Convers. Biorefin..

[30] Khir N.H.M., Fatien N., Salleh M., Salleh M.S.N., Zuhari A.M.i.A., Rosmini A.A., Zolkifli N.A.R.M. (2025). Mitigating Waterborne Health Risks Through Malachite Green Biosorption Using Tea Waste-Derived Material. J. Environ. Health..

[31] Gul S., Gul S., Gul H., Raza N., Azzouz A., Elamin M.R., Khezami L. (2025). Enhanced adsorptive removal of malachite green in environmental samples using Java plum leaves: From equilibrium to mechanism studies. Biomass Convers. Biorefin..

[32] Yildirim A., Acay H. (2025). Methylene blue and malachite green dyes adsorption onto *russula delica*/bentonite/tripolyphosphate. Heliyon.

[33] Gupta A., Dey P. (2025). Hydrothermal and NaOH-treated rice straw fibre: A potential lignocellulosic biosorbent material for removal of textile dyes from contaminated industrial wastewater. Int. J. Biol. Macromol..

[34] Kanchana S., Prakash Shyam K., Balavigneswaran C.K., Venkatesan S. (2025). High-Performance Biosorbent from Pyrolyzed Themeda arundinacea for Effective Removal of Textile Dyes: Isotherm, Kinetic, and Thermodynamic Studies. Ind. Eng. Chem. Res..

[35] Kaliaperumal K., Aboobacker J., Dhanapal V., Gayathri A., Subramanian K., Suresh A., Elumalai S., Sampath S., Devanesan S., AlSalhi M.S. (2025). Bioremediation of malachite green dye using Sargassum wightii seaweed and its biological and physicochemical characterization. Open Chem..

[36] Sudarni D.H.A., Aigbe U.O., Ukhurebor K.E., Onyancha R.B., Kusuma H.S., Darmokoesoemo H., Osibote O.A., Balogun V.A., Widyaningrum B.A., Salvestrini S. (2021). Malachite Green Removal by Activated Potassium Hydroxide Clove Leaf Agrowaste Biosorbent: Characterization, Kinetic, Isotherm, and Thermodynamic Studies. Adsorpt. Sci. Technol..

[37] Quan M.N.T., Van Lai D., Tonezzer M., Do D.Q., La D.D. (2025). Surfactant-tuned vanadium pentoxide for enhanced photocatalytic degradation of organic dyes: Nanosheet vs. microflower morphologies. RSC Adv..

[38] Abed S.H., Reshak A.H. (2025). Illuminating the Power of V_2_O_5_ Nanoparticles: Efficient Photocatalytic Degradation of Organic Dyes under Visible Light. J. Fluoresc..

[39] Karuga J., Jande Y., Kim H., King’ondu C. (2016). Fish swim bladder-derived porous carbon for defluoridation at potable water pH. Adv. Chem. Eng. Sci..

[40] Ferraz-Caetano J., Teixeira F., Cordeiro M.N.D.S. (2023). Systematic Development of Vanadium Catalysts for Sustainable Epoxidation of Small Alkenes and Allylic Alcohols. Int. J. Mol. Sci..

[41] Matusoiu F., Negrea A., Ciopec M., Duteanu N., Negrea P., Ianasi P., Ianasi C. (2022). Vanadium (V) Adsorption from Aqueous Solutions Using Xerogel on the Basis of Silica and Iron Oxide Matrix. Materials.

[42] Timoshchik O.A., Batueva T.D., Belogurova E.A., Kasikov A.G. (2024). Adsorption of Vanadium (V) on Amorphous and Modified Silica. Water.

[43] Han S., Han W., Chen J., Sun Y., Dai M., Zhao G. (2020). Bioremediation of malachite green by cyanobacterium Synechococcus elongatus PCC 7942 engineered with a triphenylmethane reductase gene. Appl. Microbiol. Biotechnol..

[44] Badawi A.K., Abd Elkodous M., Ali G.A. (2021). Recent advances in dye and metal ion removal using efficient adsorbents and novel nano-based materials: An overview. RSC Adv..

[45] Soltani A., Faramarzi M., Mousavi Parsa S.A. (2021). A review on adsorbent parameters for removal of dye products from industrial wastewater. Water Qual. Res. J..

[46] Rápó E., Tonk S. (2021). Factors Affecting Synthetic Dye Adsorption; Desorption Studies: A Review of Results from the Last Five Years (2017–2021). Molecules.

[47] Ho Y.-S., McKay G. (1999). Pseudo-second order model for sorption processes. Process Biochem..

[48] Shaikhiev I.G., Kraysman N.V., Sverguzova S.V., Spesivtseva S.E., Yarothckina A.N. (2020). Fish scales as a biosorbent of pollutants from wastewaters and natural waters (a literature review). Biointerface Res. Appl. Chem..

[49] Liang X., Zhu S., Zhong Y., Zhu J., Yuan P., He H., Zhang J. (2010). The remarkable effect of vanadium doping on the adsorption and catalytic activity of magnetite in the decolorization of methylene blue. Appl. Catal. B Environ..

[50] Avansi W., de Mendonça V.R., Lopes O.F., Ribeiro C. (2015). Vanadium pentoxide 1-D nanostructures applied to dye removal from aqueous systems by coupling adsorption and visible-light photodegradation. RSC Adv..

[51] Wei M., Evans J., Bostrom T., Grøndahl L. (2003). Synthesis and characterization of hydroxyapatite, fluoride-substituted hydroxyapatite and fluorapatite. J. Mater. Sci. Mater. Med..

[52] Azadi S., Esmkhani M., Javanshir S. (2022). New collagen-based cryogel as bio-sorbent materials for Rhodamine B removal from aqueous environments. J. Sol-Gel Sci. Technol..

[53] Mello V., Nieto-Sandoval J., Dezotti M., Sans C. (2025). Natural Pyrolusite-Catalyzed Ozonation for Nanoplastics Degradation. Catalysts.

[54] Xu B., Cui Y., A L., Zhang H., Ma Q., Wei F., Liang J. (2024). Transcriptomic and proteomic strategies to reveal the mechanism of Gymnocypris przewalskii scale development. BMC Genom..

[55] Aswathy N., Varghese J., Vinod Kumar R. (2022). Photocatalytic degradation of malachite green using vanadium pentoxide-doped NiO thin film by sol–gel spin coating. Eur. Phys. J. Plus.

[56] Mostafa E.M., Amdeha E. (2022). Enhanced photocatalytic degradation of malachite green dye by highly stable visible-light-responsive Fe-based tri-composite photocatalysts. Environ. Sci. Pollut. Res..

[57] Khezami L., Taha K.K., Ghiloufi I., El Mir L. (2016). Adsorption and photocatalytic degradation of malachite green by vanadium doped zinc oxide nanoparticles. Water Sci. Technol..

[58] Aguilar-Rosero J., Urbina-López M.E., Rodríguez-González B.E., León-Villegas S.X., Luna-Cruz I.E., Cárdenas-Chávez D.L. (2022). Development and characterization of bioadsorbents derived from different agricultural wastes for water reclamation: A review. Appl. Sci..

[59] Salehi S., Abdollahi K., Panahi R., Rahmanian N., Shakeri M., Mokhtarani B. (2021). Applications of biocatalysts for sustainable oxidation of phenolic pollutants: A review. Sustainability.

[60] Li J., Fu L., Yu Y., Yuan Y., Xi H., Wu C. (2025). Comparative Study on the Catalytic Ozonation of Biotreated Landfill Leachate Using γ-Al_2_O_3_-Based Catalysts Loaded with Different Metals. Sustainability.

